# Metformin-induced metabolic reprogramming of chemoresistant ALDH^bright^ breast cancer cells

**DOI:** 10.18632/oncotarget.1864

**Published:** 2014-03-26

**Authors:** Mario Cioce, MariaCristina Valerio, Luca Casadei, Claudio Pulito, Andrea Sacconi, Federica Mori, Francesca Biagioni, Cesare Manetti, Paola Muti, Sabrina Strano, Giovanni Blandino

**Affiliations:** ^1^ Department of Cardiothoracic Surgery, NYU Langone Medical Center, New York, NY USA; ^2^ Department of Chemistry, University of Rome ‘La Sapienza’, Rome, Italy; ^3^ Molecular Chemoprevention Group, Italian National Cancer Institute “Regina Elena”, Rome, Italy; ^4^ Translational Oncogenomic Unit, Italian National Cancer Institute “Regina Elena”, Rome, Italy; ^5^ Department of Oncology, McMaster University, Hamilton, Ontario, Canada

**Keywords:** Metformin, metabolism, chemoresistance, ALDH, metabolic reprogramming, cancer

## Abstract

Metabolic remodeling is a hallmark of cancer progression and may affect tumor chemoresistance. Here we investigated by 1H-NMR/PCA analysis the metabolic profile of chemoresistant breast cancer cell subpopulations (ALDH^bright^ cells) and their response to metformin, a promising anticancer metabolic modulator. The purified ALDH^bright^ cells exhibited a different metabolic profile as compared to their chemosensitive ALDH^low^ counterparts. Metformin treatment strongly affected the metabolism of the ALDH^bright^ cells thereby affecting, among the others, the glutathione metabolism, whose upregulation is a feature of progenitor-like, chemoresistant cell subpopulations. Globally, metformin treatment reduced the differences between ALDH^bright^ and ALDH^low^ cells, making the former more similar to the latter. Metformin broadly modulated microRNAs in the ALDH^bright^ cells, with a large fraction of them predicted to target the same metabolic pathways experimentally identified by 1H-NMR. Additionally, metformin modulated the levels of c-MYC and IRS-2, and this correlated with changes of the microRNA-33a levels. In summary, we observed, both by 1H-NMR and microRNA expression studies, that metformin treatment reduced the differences between the chemoresistant ALDH^bright^ cells and the chemosensitive ALDH^low^ cells. This works adds on the potential therapeutic relevance of metformin and shows the potential for metabolic reprogramming to modulate cancer chemoresistance.

## INTRODUCTION

It appears increasingly clear that the stable acquisition of a cancer phenotype involves metabolic remodeling. This echoes the pioneering studies from Otto Warburg and can be achieved through redirecting glucose and non glucose-dependent pathways toward anabolic generation of macromolecules, a crucial requirement for cancer cells[1, 2]. As a proof of this, multilayered modulation of metabolic enzymes by known oncogenes and tumor suppressors has been recently unveiled, with more detailed data available regarding the c-MYC-mediated modulation of glycolysis and glutamine metabolism in cancer cells [3]. Resistance to therapy is an inherent part of the pro-tumorigenic program and, almost invariably, an adverse prognostic factor for solid and non-solid tumors. Emergence within the tumor mass, of distinct chemoresistant cell populations has been recognized as an important mechanism for chemoresistance, hence tumor relapse. We and others have characterized chemoresistant cell subpopulations from breast and mesothelioma cell lines and shown that those cells are endowed with Epithelial-To-Mesenchymal (EMT) features, exhibit a precursor-like phenotype and possess high levels of Aldehyde Dehydrogenase (ALDH) activity [4, 5]. ALDH belongs to a class of detoxifying enzymes whose expression is linked to cancer chemoresistance [6, 7] and, by virtue of those high levels of ALDH activity, chemoresistant cell subpopulations can be tracked by FACS (ALDH^bright^ cells). We and others have shown that breast, ALDH^bright^-enriched cancer cell subpopulations are resistant in vitro to campthotecin, cisplatin, etoposide, topotecan [5] and docetaxel (in vivo) [8]. Tanei et al have reported that ALDH1+ cells are increased in a group of 78 breast cancer patients after neoadjuvant chemotherapy [9] and such phenomenon has been shown to occur in early passage colon cancer xenograft tumors as well [10]. Consequently, ALDH expression can be an important prognostic factor [6, 11]. Little is known regarding the metabolic features of the ALDH^bright^ chemoresistant cell subpopulations. Here we explore which are the metabolic features of the chemoresistant ALDH^bright^ cells and whether their metabolic characteristics reflect their functional properties. This may add precious knowledge to the mechanisms of tumor relapse and its modulation, to achieve anticancer effects. With regard to this latter point, metformin, an oral anti-diabetic biguanide, has been shown to target chemoresistant putative cancer stem cells from a variety of solid tumors, including lung, prostate, ovary cancer and glioma [12-14]. We and others have shown that metformin interferes with tumor engraftment and synergizes with chemotherapy in mouse xenografts, with both effects that suggest the targeting of chemoresistant, tumor initiating cell populations within the tumor mass. Additionally, we have shown a metabolic anticancer effect of metformin on unfractionated breast cancer cells lines which is partially dependent on DICER-mediated microRNA modulation [15]. However, to our knowledge, no studies have detailed the effect of metformin on purified chemoresistant cells in terms of metabolic modulation and microRNA modulation, in particular whether the metabolic effect of metformin are similar or different from those we described on unfractionated cell populations. Thus, here we studied the metabolic features of ALDH^bright^ cells isolated from three histologically different breast cancer cell lines. We first show that ALDH^bright^ cells are metabolically different from ALDH^low^ cells. Subsequently, we describe how metformin treatment affects ALDH^bright^ cell metabolism by reducing the differences between the chemoresistant ALDH^bright^ and the chemosensitive ALDH^low^ cells through targeting pyruvate metabolism, glycolysis, glutathione metabolism, pentose phosphate pathway, HIF-1α and the insulin signalling pathways. Additionally, we show that metformin treatment largely modulated the microRNA expression profile of ALDH^bright^ cells and did so by broadly modulating microRNAs predicted to impinge on cell metabolism and to target the mentioned pathways. Finally, we show that metformin modulated master cancer metabolic modulators such as c-MYC and IRS2 in the ALDH^bright^ cells and this correlated with modulation of the microRNA-33a which is known to target both the abovementioned factors. We believe this study adds on what is known on the anticancer metabolic actions of metformin and further provides a rationale for its potential use combined with chemotherapy to prevent tumor relapse, through targeting of chemoresistant, residual cell subpopulations.

## RESULTS

### FACS-based isolation of breast cancer ALDH^bright^ and ALDH^low^ cells (Fig.1)

**Fig.1:**
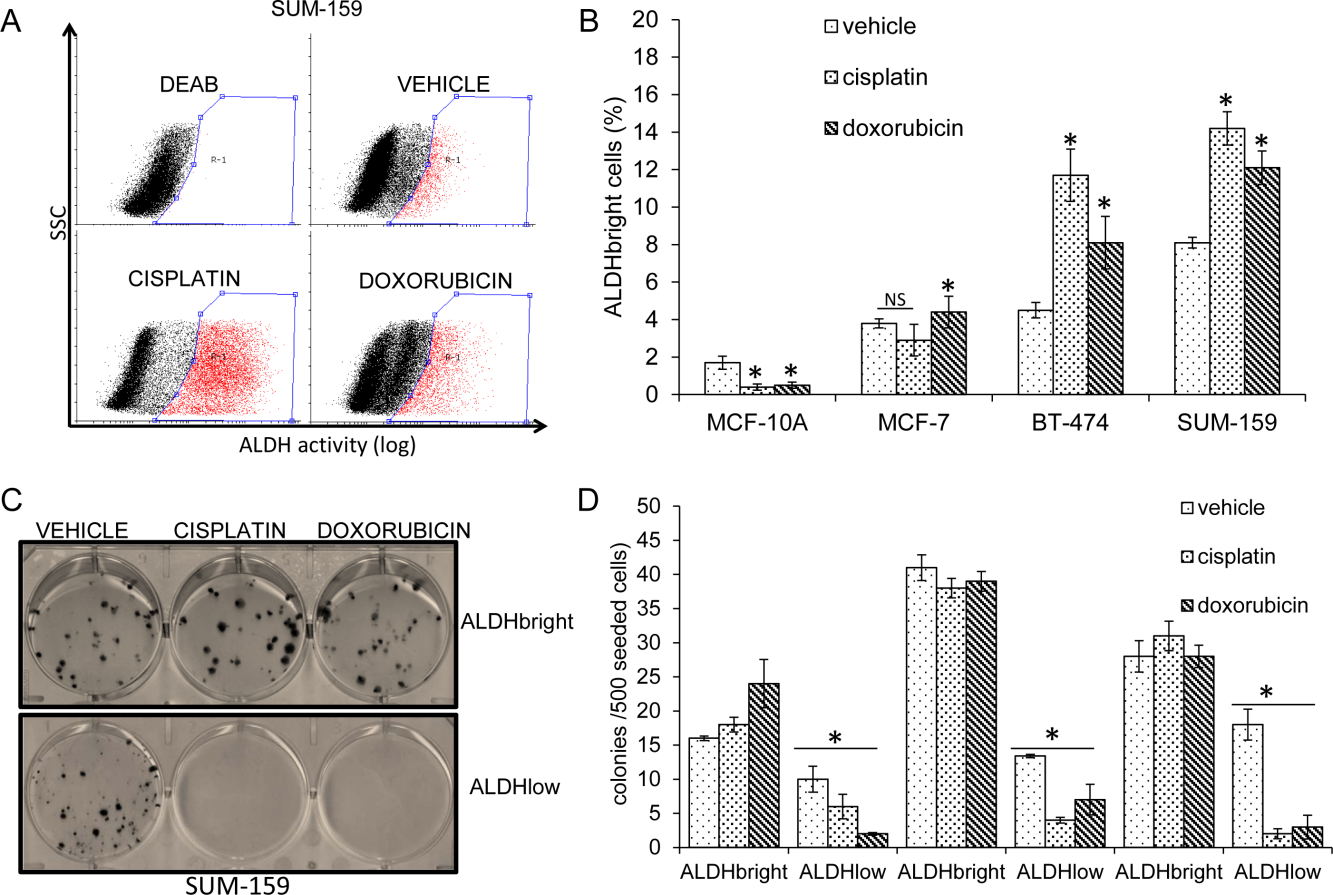
Breast cancer ALDHbright cells are chemoresistant A. Representative FACS dot plots of SUM-159 cells assayed for Aldhehyde Dehydrogenase (ALDH) activity. Cells enriched for ALDH activity (ALDH^bright^) were gated (red-r1). To set the background, the percentage of ALDH^bright^ cells was determined in the presence of DEAB, an inhibitor of the ALDH enzyme (upper left plot). B. Histogram showing the percentage of ALDH^bright^ cells in the indicated cell lines treated with vehicle, cisplatin or doxorubicin for 72hrs. Mean ± SE values of two independent experiments were reported. NS: not significant. *: p<0.05 (when compared to vehicle-treated samples). Additional representative dot plots relative to the graph in 1B are available in Suppl. Fig. 1. C. Clonogenic assay. Representative micrographs of the colonies formed by SUM-159 cells pulsed with vehicle, cisplatin and doxorubicin for 16hrs and seeded at clonal density. Colonies stained with crystal violet 9 days later. D. Histogram showing the absolute number of colonies formed by the indicated cell lines treated as in 1C. Please note that the untransformed MCF-10A were not assayed because non clonogenic. Mean ± SE values of two independent experiments were reported. *: p<0.05 (as compared to ALDH^bright^ cells).

We determined by FACS the percentage of cells endowed with high ALDH activity (ALDH^bright^ cells) from MCF-7, BT-474 and SUM-159 cell lines. ALDH enzymatic activity was detected by following the accumulation of a fluorescent ALDH substrate, with the mean fluorescence of cells pretreated with a known inhibitor of the ALDH activity (DEAB) set as background (Fig 1A-B). We found that all the breast cancer cell lines in our collection contained readily detectable ALDH^bright^ cells, although with some variability among each cell line. Given the involvement of the ALDH^bright^ cells in mediating cancer chemoresistance, we treated MCF-7, BT-474 and SUM159 cells with cisplatin and doxorubicin, two commonly used chemotherapy agents for breast cancer and we evaluated the percentage of ALDH^bright^ cells among the surviving cell populations. We found that, upon chronic treatment with cisplatin (40μM, 72hrs) or doxorubicin (0.1μM, 72hrs) the percentage of breast cancer ALDH^bright^ cells increased in BT-474 and SUM-159 cells and remained unchanged or only slightly decreased in the MCF-7 cells (Fig. 1A-B and Suppl. Fig.1). Interestingly, a barely detectable number of ALDH^bright^ cells was found in the dysplastic, non-transformed breast cells MCF10A (1.4 ± 0.4%)(Fig.1B and Suppl. Fig.1). This is in agreement with previous work [5]. However, the MCF-10A ALDH^bright^ cells became undetectable upon cisplatin and doxorubicin treatment (Fig.1B and Suppl. Fig.1), in agreement with the chemosensitivity of the cell line[16]. Clonogenic assays with FACS-purified ALDH^bright^ and ALDH^low^ cells confirmed that the ALDH^bright^ cells in the transformed breast cancer cell lines represented the main chemoresistant cell subpopulation as compared to the ALDH^low^ cells (the latter representing most of the cells in unsorted cell lines) (Fig. 1C-D). The availability of the described experimental system prompted us to characterize the metabolic profile of the purified ALDH^bright^ chemoresistant cell subpopulations.

**Table 1:** Metabolites contributing to the difference between ALDH^bright^ and ALDH^low^, determined by the analysis of O-PLS-DA loadings.

Metabolite	ALDH^bright^ vs. ALDH^low^*	Related KEGG Pathway Maps**
3-Hydroxy-butyrate	High (p)	Synthesis and degradation of ketone bodies
Alanine	High (p)	Alanine, aspartate and glutamate metabolism
Lactate	High (p)	Glycolysis/Gluconogenesis
		Pyruvate metabolism
Acetate	Low (p)	Glycolysis/Gluconogenesis
		Pyruvate metabolism
Succinate	Low (c)	Citrate cycle (TCA cycle)
		Oxidative phosphorylation
		Alanine, aspartate and glutamate metabolism
		Tyrosine metabolism
		Phenylalanine metabolism
		Carbon metabolism
Glucose	Low (c)	Glycolysis/Gluconogenesis
		Pentose phosphate pathway
		Amino sugar and nucleotide sugar metabolism
		HIF-1 signaling pathway
		Insulin signaling pathway
Formate	High (p)	Pyruvate metabolism
		Carbon metabolism

* “High” indicates that the metabolite was at a higher concentration in medium samples from ALDH^bright^ cells; “Low” indicates that the metabolite was at a lower concentration in medium samples from ALDH^bright^ cells; “c” and “p” for each metabolite indicate consumption or production, respectively.

** The metabolites are mapped to their respective biochemical pathways as delineated in the Kyoto Encyclopedia of Genes and Genomes (Release 69.0, January 1, 2014; KEGG, http://www.genome.jp/kegg).

### The metabolic profile of ALDH^bright^ cells is different from that of ALDH^low^ cells (Fig. 2)

**Figure 2:**
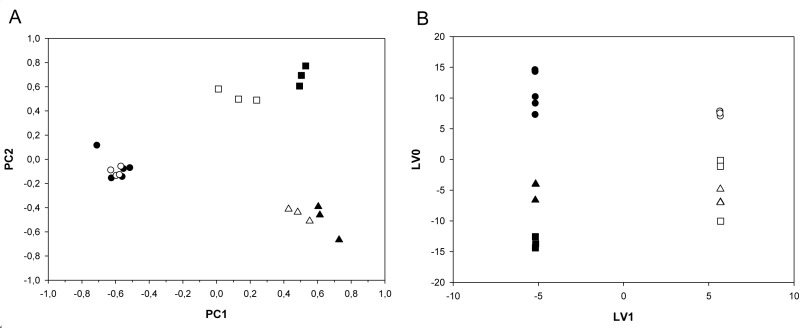
A-B ALDH^bright^ and ALDH^low^ cells are metabolically distinguishable cell subpopulations Overview of the PCA (A) and OPLS-DA (B) models built on the NMR datasets of medium samples collected from ALDH^low^ (*open*) and ALDH^bright^ (*filled*) populations of MCF-7 (*triangle*), BT-474 (*square*) and SUM-159 (*circle*) cell lines. A. The obtained PCA model of ALDH subpopulations was dominated by the effect of cell line differences acting as major order parameter: PC1 and PC2 components are mainly responsible for among cell lines differences (see also Table 1). B. OPLS-DA analysis of the same samples as from A shows a clear separation between the ALDH^low^ and ALDH^bright^ populations, independently from cell type, was found in the first component.

The conditioned media from FACS sorted ALDH^bright^ and ALDH^low^ MCF-7, BT-474 and SUM-159 cells, were analysed using ^1^H-NMR spectroscopy. NMR profiles identified at the beginning of observation were compared to those representing the end of the experiment (after 24 hours). In this way the resulting positive net balances indicated release or production of metabolites, whereas negative net balances indicated uptake or consumption of metabolites. We analyzed the NMR data by using Principal Component Analysis (PCA) carried out on untreated samples. This unsupervised method allows orthogonal decomposition of variance associated with the analyzed metabolites. Six components (PC 1-6) were sufficient to explain 65% of total variability of the system. In Fig. 2A, the PC1 (the most important metabolic component explaining 21% of total variance of the samples) and PC2 (the second most important metabolic component explaining 13% of total variance of the samples) discriminated between the conditioned media of the BT-474, MCF-7 and SUM-159-PT cell lines. Being the three breast cancer cell lines histologically and biologically very different, this was partially expected. To a minor extent, the PC1/PC2 plane was also capable of discriminating the subpopulation state (ALDH^bright^ or ALDH^low^). To detail more the previous observations we carried out an ANOVA test on the component scores relative to the BT-474, MCF-7 and SUM-159-PT samples by establishing, as sources of variation, the cell subpopulation (ALDH^bright^ and ALDH^low^ cells) and the cell line (MCF-7, BT-474 and SUM-159-PT) (Suppl. Table 1).This analysis showed that the cell line differences acted as a major factor affecting the distribution of the samples (F statistics = 98.42 and 432.77 for PC1 and PC2, respectively). However, the difference among the cell subpopulations (ALDH^bright^ vs ALDH^low^) common to all cell lines was detectable by the PC1 and PC4 components (F statistics = 35.54 and 28.08 for PC1 and PC4, respectively). The ALDH^bright^ vs ALDH^low^ difference, cell line dependent, was detectable by the PC1 and PC3 components (F statistics = 20.76 and 18.39 for PC1 and PC3, respectively). The detectable impact of the ALDH^bright^ vs ALDH^low^ condition on the whole metabolic profile of the samples analyzed prompted us to exclude the major effect of the cell line and to focus on the differences between ALDH^bright^ and ALDH^low^ cell samples by using the orthogonal projections to latent structures-discriminant analysis (OPLS-DA) (Fig. 2B). The OPLS-DA model generated one Latent Variable (LV) that explained 55% of the X-variance and 100% of Y-variance with a cumulative predicted fraction of the joint X and Y variation of 85%. A clear separation between the ALDH^low^ and ALDH^bright^ populations was found in the first component (LV1) independently from cell type (while the metabolic difference between the cell lines were observed on LV0) (Fig. 2B). From the analysis of the O-PLS-DA loadings we observed that 3-hydroxy-butyrate, alanine, lactate, acetate, succinate, glucose and formate were the main discriminatory metabolites responsible for the difference between ALDH^bright^ and ALDH^low^ cell subpopulations (Table 1).

### Metformin-induced changes in the metabolic phenotype of ALDH^bright^ and ALDH^low^ cells (Fig. 3)

**Figure 3A-C:**
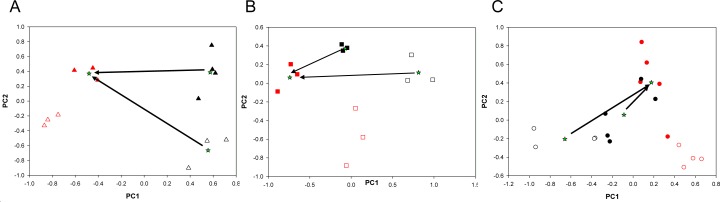
PCA analysis of vehicle- and metformin-treated ALDH^bright^ and ALDH^low^ cells PCA models built on the NMR datasets of medium samples collected from ALDH^low^ (*red*) and ALDH^bright^ (black) subpopulations of vehicle- (*open*) and metformin-treated (*filled*) MCF-7 (*triangle*), BT-474 (*square*) and SUM-159(*circle*) cells). Please note that filled samples (metformin-treated cells) occupy a very similar location in the component space. In each line, the Euclidean distance (arrows) between the centroids (stars) of the two treated subpopulations is smaller than the distance between the treated ALDH^low^ and untreated ALDH^bright^ cells, suggesting that the metabolism of ALDH^bright^ and ALDH^low^ cells became more similar upon metformin treatment.

Next, we tested the effect of metformin on the metabolic processes of the ALDH^bright^ and ALDH^low^ cell subpopulations. More specifically, we detailed the ^1^H-NMR profile of the conditioned media from the ALDH^bright^ and ALDH^low^ subpopulations of BT-474, MCF-7 and SUM-159 cells treated with vehicle (PBS1X) or metformin at a non cytotoxic concentration/length of treatment combination (0.5mM-24hrs) (Suppl. Fig. 2). This was done to avoid interference of apoptotic processes with the 1H-NMR analysis.

PCA analysis of the vehicle- and metformin- treated ALDH^bright^ and ALDH^low^ cells from all three breast cancer cell lines was very complex and revealed, again, a predominant effect of the differences among the parental cell lines (thus masking the effect of metformin treatment)(Suppl. Table 2). Therefore, we analyzed the effect of metformin treatment on the ALDH^bright^ and ALDH^low^ cell subpopulations for each cell line independently. The PCA results for each dataset are depicted in Figure 3A-C, where the score plots for the first two model components are shown. A clear separation among control and metformin-treated samples for each subpopulation was obtained. This revealed that the PC1 (for the BT-474 and SUM-159 cells), and the PC2 (for the MCF-7) were mainly responsible for the differences between vehicle- and metformin-treated ALDH^bright^ and ALDH^low^ cell subpopulations. The quantitative PCA approach revealed that, when considering the same metabolic process for both cell subpopulations, the effects of metformin were more evident in the ALDH^bright^ as compared to the ALDH^low^ cells. The maximum difference between the metformin- and vehicle-treated groups (thus, the maximum drug effect) was observed for the BT-474 and SUM-159 ALDH^bright^ cells on the PC1 and for the MCF-7 ALDH^bright^ cells on the PC2 (Suppl. Table 3). Notably, we found that the treatment with metformin reduced the difference between ALDH^bright^ and ALDH^low^ cells. More specifically, we found that, for each cell line, the Euclidean distance (arrows) between the centroids (i.e, the barycenter of the group of samples)(stars) (Fig. 3A-C) of the treated ALDH^low^ and ALDH^bright^ samples was smaller than the distance between the treated ALDH^low^ and the untreated ALDH^bright^ samples, suggesting that Metformin changed the metabolism of ALDH^bright^ towards a phenotype more similar to that of metformin-treated ALDH^low^ cells This was very evident for the SUM-159 cell line.

### Metabolomic signatures of Metformin treated ALDH^bright^ cells (Fig. 4)

**Figure 4:**
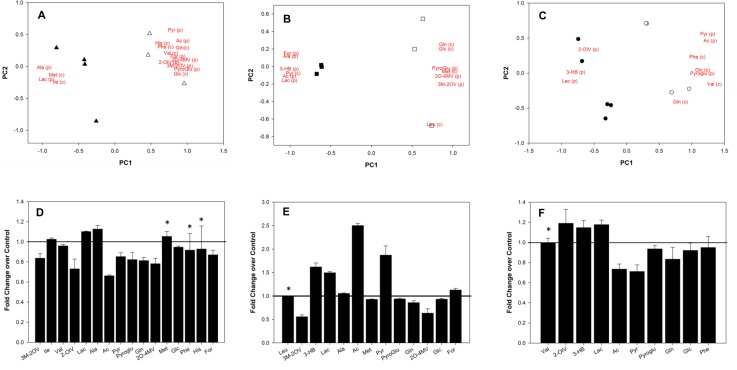
A-F: Metabolomic analysis of metformin treated ALDH^bright^ cells Upper panels. Overview of the PCA model built on the NMR dataset of media samples of control (*open*) and metformin-treated (*filled*) ALDH^bright^ cells from MCF-7 (*triangle*), BT-474 (*square*) and SUM-159 (*circle*) cells. The score and loading plots of the first two components (PC1 *versus* PC2) are shown superimposed. The score plot shows the differentiation between untreated and metformin-treated samples, while the loading plot highlights which metabolites are responsible in separating control and metformin-treated samples. Lower panels. Histograms indicate the relative levels of the metabolites considered in the score/loading plots for MCF-7 (right), BT-474 (middle) and SUM-159 (left). Loading values are represented using the abbreviation of metabolites: leucine, Leu; valine, Val; 3-Methyl-2-oxovalerate, 3M-2OV; 3-hydroxy-butyrate, 3-HB; lactate, Lac; alanine, Ala; acetate, Ac; methionine, Met; glutamine, Gln; glutamate, glu; pyruvate, Pyr; pyroglutamate, Pyroglu; 2-Oxo-4-methylvalerate, 2O-4MV; glucose, Glc; histidine, His, phenylalanine, Phe;formate, For.

Next, we detailed the metabolic profiles relative to the vehicle- and metformin- treated ALDH^bright^ cells (Fig. 4A-E). For each cell line, the PCA produced solutions with two significant components, explaining about 52%, 65% and 47% of the total variability of the system for the MCF-7, BT-474, and SUM-159 cells, respectively (data not shown). This highlighted significant differences between the two groups on the PC1. Additionally, despite some differences between the cell lines, we observed similarities in the correlation patterns of metabolite loadings (Fig. 4A-E). For each cell line, the PC1 included the following variables with the highest correlation levels: glutamine and glucose consumption and pyroglutamate production with positive loadings as well as lactate production with negative loadings. Therefore, the PC1 analysis indicated that metformin treatment induced higher consumption of glutamine and glucose as well as higher production of lactate (positive correlation with PC1) compared to untreated cells (Fig. 4C-E).

The negative correlation between glutamine and glucose consumption and lactate production in the loading plots of the ALDH^bright^ cells from all the analysed cell lines suggested higher fluxes through glycolysis or glutaminolysis in the treated cells compared to untreated controls. Previous studies using NMR analysis with [1,2-^13^C]-glucose revealed that, in metformin-treated cells, lactate is more produced from glutaminolysis rather than from glycolysis therefore suggesting that the net effect of metformin consists of a reduction of the glycolytic flux. A lower production of pyroglutamate upon metformin treatment was also observed (negative correlation with PC1). A lower excretion of pyroglutamate suggested a reduced level of intracellular glutathione. In facts, pyroglutamate, also known as 5-oxoproline, is converted to glutamate by 5-Oxoprolinase. As glutamate is required in the first step of GSH synthesis, the lower production of pyroglutamate observed in treated cells suggest a minor level of intracellular glutathione. Moreover, in MCF-7 and BT-474 cell lines, we observed that glucose and glutamine consumption correlated also with the production of alanine (opposite loadings) suggesting a higher activation of alanine aminotransferases in the metformin-treated cells. However, the fact that acetate was a strong negative loader on PC1 for BT-474, demonstrated that the alanine aminotransferase pathway was also used to provide precursors needed for fatty acid synthesis. Nevertheless, the higher excretion of acetate into media of the metformin-treated cells reflected the smaller availability of acetyl-CoA units for fatty acid synthesis. For the MCF-7 and BT-474 cells, the negative correlation of 3-methyl-2-oxovalerate and 2-oxo-4-methylvalerate loadings within the metformin-treated cells as opposed to the control-treated cells indicated a higher metabolic flux through the branched-chain amino acid aminotransferase pathway. This strongly suggests the use of branched amino acids for energy production instead of its use for macromolecule biosynthesis in the metformin-treated cells. Analysis of the identified metabolites with the KEEG pathways indicated that, in all three ALDH^bright^ cell subpopulations treated with metformin, we observed perturbations of the glycolysis, pyruvate metabolism, glutathione metabolism, purine and pyrimidine metabolism, alanine, aspartate, glutamate, arginine and proline metabolism, pentose phosphate pathway, amino sugar and nucleotide sugar metabolism, HIF-1α and the insulin signalling pathways in all cell lines (Table 2).

**Table 2:** Metabolic pathways perturbed by Metformin in all the analyzed breast cancer cell lines

Metabolite	Metformin vs. control*	Related KEGG Pathway Maps**
Lactate	High (p)	Glycolysis/Gluconogenesis
		Pyruvate metabolism
Pyroglutamate	Low (p)	Glutathione metabolism
Glutamine	Low (c)	Purine metabolism
		Pyrimidine metabolism
		Alanine, aspartate and glutamate metabolism
		Arginine and Proline metabolism
Glucose	Low (c)	Glycolysis/Gluconogenesis
		Pentose phosphate pathway
		Amino sugar and nucleotide sugar metabolism
		HIF-1 signaling pathway
		Insulin signaling pathway

* “High” indicates that the metabolite was at a higher concentration in medium samples from metformin-treated cells; “Low” indicates that the metabolite was at a lower concentration in medium samples from metformin-treated cells; “c” and “p” for each metabolite indicate consumption or production, respectively.

** The metabolites are mapped to their respective biochemical pathways as delineated in the Kyoto Encyclopedia of Genes and Genomes (Release 69.0, January 1, 2014; KEGG, http://www.genome.jp/kegg).

**Figure 5:**
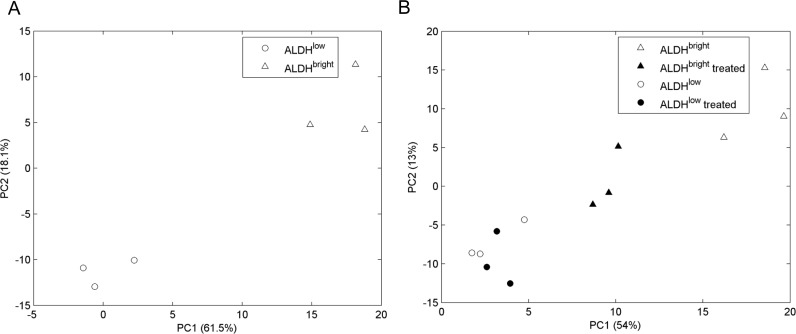
A. ALDH^bright^ and ALDH^low^ cells differ in their microRNA expression profile. PCA of the microRNA expression profile of the untreated ALDH^bright^ and ALDH^low^ cell subpopulations derived from the SUM-159 cells (497 microRNAs expressed/analyzed). B. Metformin modulates microRNAs in the ALDH^bright^ and ALDH^low^ cells. PCA of 125 miRNAs modulated by metformin in ALDH^bright^ and ALDH^low^ SUM-159 cells. Principal Component Analysis. Percentage of the explained variance is indicated for the first two components.

**Figure 6:**
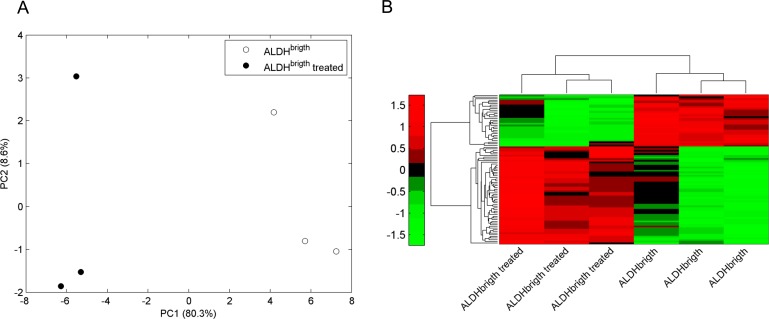
Metformin modulates microRNAs associated with metabolic functions in the ALDH^bright^ cells A. PCA of 89 miRNAs modulated by metformin and involved in metabolic pathways in SUM-159 cells. Principal Component Analysis. Percentages of the explained variance is indicated for the first two components. B. Heat Map of 89 miRNAs modulated by metformin and involved in metabolic pathways. Unsupervised Hierarchical Clustering. Red indicates higher expression and green lower expression of the indicated miRNAs for each sample. The list of the 89 microRNAs used for the heat map can be found in Suppl. Table 4.

Metformin-mediated modulation of microRNAs sustains its metabolic effects on the ALDH^bright^ cells (Fig. 5 and 6). We previously demonstrated that metformin exerts anticancer metabolic effect on unsorted breast cancer cell populations by broadly modulating the metabolic pathways, at least partially in a DICER-dependent microRNA modulation[15]. We verified whether a similar mechanism would take place in the sorted ALDH^bright^ cells and whether this may explain the metabolic changes observed in the treated cells. Therefore, we analysed the microRNA expression profile of vehicle- and metformin- treated, FACS sorted ALDH^bright^ cells (Fig. 5). This first revealed that, at steady state, the microRNA expression profile of ALDH^low^ and ALDH^bright^ was very different, mirroring the observed difference in their metabolic profile. In facts, the unsupervised PCA analysis of 497 expressed microRNAs in the ALDH^low^ and ALDH^bright^ cell subpopulations revealed a clear separation of the two cell subpopulations on both the PC1 and the PC2 (61.5% and 18.1% of the total variance, respectively)(Fig.5A). Next, we evaluated the microRNA expression profile of vehicle- and metformin- treated ALDH^bright^ and ALDH^low^ cells derived from the SUM-159 cells. This provided us with two observations: first, metformin treatment induced changes in microRNA expression levels in both ALDH^bright^ and ALDH^low^ cells (Fig.5B), with a slightly more evident effect on the ALDH^bright^ cells; second, metformin treatment reduced the distance in the microRNA expression profile between ALDH^bright^ and ALDH^low^ cells (Fig. 5B). This was reminiscent of the metabolic effects of the drug on the ALDH^bright^ and ALDH^low^ cells, which were consistent with the acquisition of a more similar metabolic profile between the two cell subpopulations. Altogether, these observations suggest that metformin treatment of breast cancer ALDH^bright^ cells partially reverted a chemoresistant and clonogenic phenotype to a chemosensitive, more differentiated one. In order to support this observation, we detailed the microRNA modulation in the metformin treated-ALDH^bright^ cells (Fig.6A) Interestingly, a large fraction (89/125) of the microRNAs significantly modulated in the metformin-treated ALDH^bright^ cells (Suppl. Table 4) was predicted to target metabolic pathways (Table 3) and caused a strong separation of the samples on the PC1 (Fig. 6A) We found that the pathways found deregulated by metformin in the ^1^H-NMR studies were represented within those predicted by the microRNA analysis, thus establishing a correlation between the effect of metformin on the microRNAs and its metabolic activity(Table 3).

**Table 3:** Main metabolic functions predicted to be modulated by the metformin-regulated microRNAs

insulin path	miR-335-5p,miR-124-3p,miR-98,miR-16-5p,miR-155-5p,miR-26b-5p,miR-1,miR-7-5p,miR-21-5p,let-7b-5p,miR-192-5p,miR-128,miR-148b-3p,miR-375,miR-30a-5p,miR-130b-3p,miR-125a-5p,miR-103a-3p,miR-93-5p,miR-10a-5p,miR-34a-5p,miR-24-3p,miR-19b-3p,miR-193b-3p,miR-122-5p,miR-186-5p,let-7,miR-126-3p,miR-181a-5p,miR-29c-3p,miR-101-3p,miR-141-3p,miR-145-5p,miR-214-3p,miR-590-3p,miR-33a-5p,miR-769-5p,miR-200a-3p,miR-32-5p,miR-132-3p,miR-27a-3p,miR-149-3p,miR-217,miR-200c-3p,mir-199a,miR-615-3p,mir-199a*,miR-22-3p,miR-374b-5p,miR-429,miR-9-5p,miR-140-3p,miR-18a-5p,miR-200b-3p,miR-421,miR-96-5p'
alanine aspartame metabolism	'miR-335-5p,miR-155-5p,miR-16-5p,miR-26b-5p,miR-34a-5p,miR-192-5p,miR-128,miR-24-3p,miR-27a-3p,miR-21-5p,miR-1,miR-124-3p,miR-590-3p,miR-101-3p,miR-96-5p,miR-186-5p,miR-30b-5p,miR-7-5p,miR-183-5p,miR-98,miR-32-5p'
amino sugar metabolism	'miR-26b-5p,miR-124-3p,miR-155-5p,miR-30a-5p,miR-375,miR-30b-5p,miR-1,miR-34a-5p,miR-335-5p,miR-106b-5p,mir-30,miR-98,miR-16-5p,miR-32-5p,miR-9-5p,let-7b-5p,mir-199a,miR-21-5p,miR-101-3p,miR-193b-3p,miR-128,miR-590-3p,miR-24-3p,miR-192-5p,miR-148b-3p,miR-122-5p'
Aminoacyl-trna biosinthesis	'miR-16-5p,miR-155-5p,miR-26b-5p,let-7b-5p,miR-30a-5p,miR-93-5p,miR-101-3p,miR-124-3p,miR-19b-3p,miR-192-5p,miR-1,miR-21-5p,miR-98,miR-130b-3p'
cysteine metabolism	'miR-26b-5p,miR-375,miR-193b-3p,miR-155-5p,miR-335-5p,miR-29b-3p,let-7b-5p,miR-29c-3p,miR-369-5p,miR-34a-5p,miR-29a-3p,miR-16-5p,miR-33a-5p,miR-191-5p,mir-199a,mir-30,miR-186-5p,miR-548b-3p,miR-30a-5p,miR-152,let-7d-5p,miR-106b-5p,mir-148,miR-1,miR-148b-3p,miR-7-5p,miR-192-5p,miR-124-3p'
D-glutamine and glutamate	'miR-335-5p,miR-7-5p'
glycolisis	'miR-335-5p,miR-124-3p,miR-155-5p,miR-30a-5p,mir-17-92,miR-375,miR-34a-5p,miR-1,miR-7-5p,miR-16-5p,miR-26b-5p,mir-199a,miR-122-5p,mir-30,miR-148b-3p,let-7b-5p,miR-98,miR-24-3p,miR-22-3p,miR-132-3p,miR-145-5p,miR-133b,miR-192-5p,miR-181a-5p,miR-33a-5p,miR-133a,miR-27a-3p,miR-128,miR-9-5p'
pentose phospate pathway	'miR-124-3p,miR-335-5p,miR-1,miR-26b-5p,miR-148b-3p,miR-375,miR-34a-5p,edited-hsa-mir-376a-5p,mir-1b,let-7b-5p,miR-192-5p,mir-30,miR-142-3p,miR-30a-5p,miR-373-3p'
Pyruvate metabolism	'miR-335-5p,miR-34a-5p,miR-155-5p,miR-26b-5p,let-7b-5p,miR-16-5p,miR-30a-5p,miR-124-3p,miR-24-3p,miR-7-5p,miR-1,miR-33a-5p,miR-133b,miR-27a-3p,miR-375,miR-133a,miR-21-5p,miR-22-3p,miR-122-5p,miR-23b-3p,miR-193b-3p,miR-148b-3p,miR-98,miR-192-5p,miR-181a-5p'
‘purine’ metabolism	'miR-124-3p,miR-335-5p,let-7b-5p,miR-26b-5p,miR-98,miR-155-5p,miR-30a-5p,miR-1,miR-16-5p,miR-193b-3p,miR-7-5p,miR-34a-5p,miR-192-5p,mir-30,miR-24-3p,miR-96-5p,miR-128,miR-375,miR-19b-3p,miR-21-5p,miR-103a-3p,miR-196a-5p,miR-148b-3p,let-7d-5p,miR-142-3p,miR-93-5p,miR-133b,miR-186-5p,edited-hsa-mir-376a-5p,miR-130b-3p,miR-133a,miR-9-5p,miR-421,miR-122-5p,mir-132/mir-212,miR-340-5p,miR-18a-5p'
‘pyrimidine’ metabolism	'miR-26b-5p,let-7b-5p,miR-34a-5p,miR-124-3p,miR-155-5p,miR-193b-3p,miR-192-5p,miR-1,miR-7-5p,miR-335-5p,miR-30a-5p,miR-16-5p,miR-24-3p,miR-98,mir-30,miR-186-5p,miR-148b-3p,miR-375,miR-21-5p,miR-196a-5p,miR-101-3p,miR-128,miR-103a-3p,miR-142-3p,miR-122-5p,miR-421,miR-9-5p,miR-10a-5p,mir-199a*,miR-96-5p,miR-590-3p,let-7d-5p,mir-132/mir-212,miR-374b-5p'
Sinthesis of ketone Bodies	'miR-21-5p,miR-192-5p,miR-18a-5p,miR-1,miR-335-5p,miR-23b-3p,miR-155-5p,miR-19b-3p,miR-26b-5p,miR-186-5p,miR-375,miR-124-3p,miR-96-5p'
‘valine metabolism’	'miR-124-3p,miR-26b-5p,miR-192-5p,miR-1,miR-155-5p,miR-34a-5p,miR-335-5p,miR-16-5p,miR-32-5p,miR-193b-3p,miR-21-5p,miR-9-5p,miR-19b-3p,miR-128,let-7b-5p,miR-186-5p,miR-96-5p,miR-7-5p,miR-18a-5p,miR-27a-3p,miR-23b-3p'

**Figure 7:**
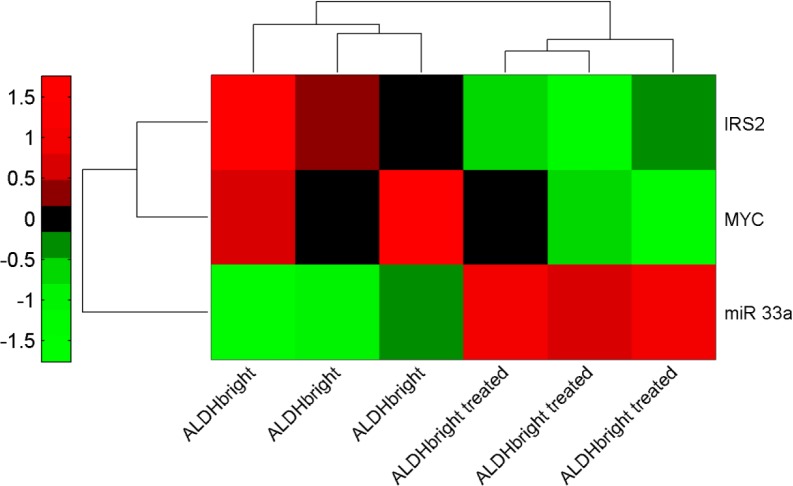
Metformin inversely modulates mir-33a and its targets The heat map shows opposite regulation of the microRNA-33a and its gene targets (c-MYC, IRS-2)(Normalized intensity values) in metformin treated ALDH^bright^ cells. Triplicate experiments.

**Figure 8:**
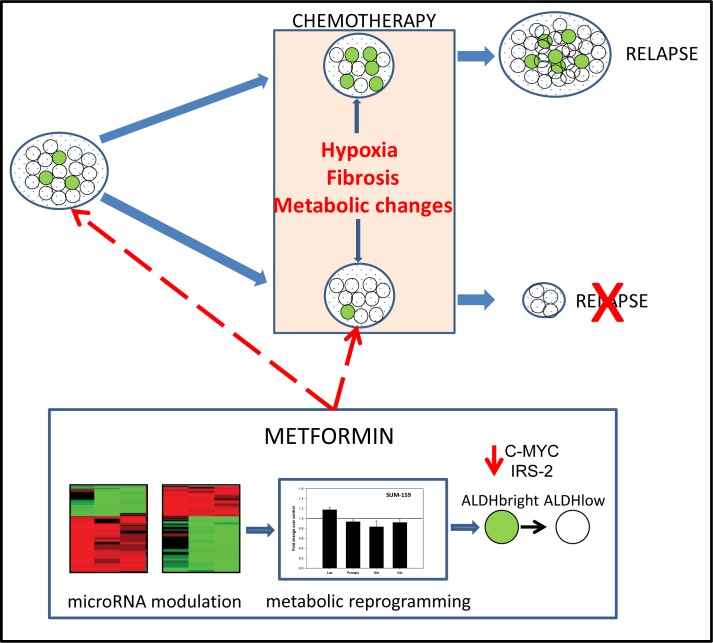
Representative working model Metformin modulates the microRNA profile of ALDH^bright^ cells thereby impacting on their metabolic properties. This makes the ALDH^bright^ cells more similar to the ALDH^low^ cells and affect chemoresistance of the tumor, potentially reducing its relapse. Metformin proposed action may take place either before or in conjunction with chemotherapy. Dashed arrows indicate speculated actions of the drug.

Metformin targets c-MYC and IRS-2 via mir-33a modulation (Fig. 7). We and others have recently demonstrated that the anticancer metabolic activity of metformin toward unsorted breast cancer cells is partially due to a mir-33a-dependent modulation of c-MYC levels [15]. In facts, C-MYC is a central modulator of cancer cell metabolism, alone or in cooperation with HIF-1α [17, 18]. To assess whether a similar mechanism would operate in the ALDH^bright^ cancer cells as well, we evaluated the levels of microRNA 33a and its target c-MYC in ALDH^bright^ cells, treated with vehicle or metformin, respectively. By performing quantitative PCR, we observed a strong modulation of mir-33a levels which anti-correlated with those of c-MYC. Additionally, we found that the levels of IRS-2, a central modulator of insulin signalling in normal and cancer cells and a target of microRNA33a as well [19] were modulated by metformin (Fig. 7). A working model that generally resumes the collected observations is shown in Fig. 8 (Fig. 8).

## DISCUSSION

We have recently shown that a complex interplay of microRNA modulation and pathway signalling underlies the anticancer effects of metformin, an anti-diabetic agent which epidemiological evidence suggest to play an important role in cancer prevention [15, 20, 21]. With the aim of detailing our previous studies, in this work we have studied the metabolic changes taking place in breast ALDH^bright^ cells, the main cell subpopulation responsible for the chemoresistance of breast tumors. This because understanding the metabolism of chemoresistant cell subpopulations may aid the identification of the basic mechanism sustaining their chemoresistance, a major factor shaping the prognosis of breast cancer patients. Our experimental systems included three breast cancer cell lines, differing for histological origin and growth rate. Indeed, our metabolomics analysis identified the cell line specificity as a major determinant of the differences between the samples, thus confirming the efficiency of the approach. Despite this, the OPLS-DA analysis allowed us to focus on the differences between vehicle- and metformin- treated ALDH^bright^ cells. First, we found that the metabolic profile of the purified ALDH^bright^ cell subpopulations was significantly different from that of the ALDH^low^ cells. When focusing on the metabolic changes induced by metformin treatment and common to the ALDH^bright^ cells from all the cell lines examined (MCF-7, BT-474, SUM-159-ALDH^bright^ cells), an increase in glucose and glutamine uptake and lactate production as well as the decrease in pyroglutamate production were observed upon metformin treatment. Interestingly, a reduced excretion of pyroglutamate from the metformin-treated cells indicated reduced levels of intracellular glutathione. This is in line with recent findings showing that metformin affected the glutathione (GSH) homeostasis of breast cancer cells, with the reduced glutathione biosynthesis correlating with a blockage of de novo purine/pyrimidine synthesis[22]. Our observations suggest that negative modulation of the glutathione homeostasis in the ALDH^bright^ cell subpopulations may underlie the anticancer action of metformin. In facts, higher levels of intracellular glutathione have been described in chemo- and radio-resistant “cancer stem cell –like (CSCs)” cell subpopulations derived from MMTV-WNT1 breast tumors[23]. Additionally, this correlated with a broad modulation of the mRNA levels of the genes involved in the glutathione metabolism. We speculate that metformin treatment may reduce the glutathione pool in the breast ALDH^bright^ cell subpopulations thereby abating their resistance to therapy. Interestingly, recent findings suggest that blockage of c-MYC downstream effectors affected glycolysis and glutathione biosynthesis in MYC-driven mouse lymphoma models[24]. In line with this, we have found that metformin downregulated the c-MYC levels in ALDH^bright^ cells, possibly by upregulating the mir-33a levels. C-MYC activation in ALDH^bright^ cells may strongly cooperate with the activation of HIF-1 metabolic effectors, such as several glycolytic enzymes [3, 25, 26]. The latter phenomenon was observed by our ^1^H-NMR analysis. Additionally, IRS-2 is downregulated by metformin and, like for c-MYC, this inversely correlated with the levels of mir-33a in the metformin treated cells. IRS-2 is an important modulator of aerobic glycolysis in mouse mammary cancer cells [27]. Notably, modulation of c-MYC and IRS-2 mRNA levels was already shown by us in unfractionated, metformin treated breast cells lines [15]. What we believe is remarkable here is that the treatment with metformin drastically reduced the metabolic difference between ALDH^bright^ and ALDH^low^ cells. A very similar effect was observed on the microRNA expression profile of metformin treated ALDH^bright^ and ALDH^low^ cells. This is very interesting since the ALDH^low^ cells represent the chemosensitive fraction of solid tumors and it indicates the potential of metformin for metabolic reprogramming of chemoresistant cell subpopulations. This represents a further mechanism of cancer interference by this promising compound (Fig. 8).

## METHODS

### Reagents

Metformin (1,1-Dimethylbiguanide-hydrochloride) Cisplatin and and Doxorubicin-hydrochloride were dissolved according to the manufacturer's instructions. (Sigma, St Louis, MO, USA)

### Cell culture conditions

MCF-7 and BT-474 breast cancer cell lines were grown in DMEM/F12 supplemented with 10% non-heat inactivated FBS (Invitrogen-GIBCO, Carlsbad, CA, USA); the MCF10A and SUM159 cells were grown in DMEM/F-12 supplemented with 5% FBS, Insulin 5μgr/ml (SIGMA) and Hydrocortisone 0.5 μgr/mL (SIGMA).

### ALDH activity assay

ALDH activity was detected by FACS CALIBUR instrument (BD Biosciences, Franklin Lakes, New Jersey, USA). ALDEFLUOR kit (Stem Cell Technologies, Vancouver, Canada) was used according to the manufacturer's instructions. ALDH^bright^ cells were defined as the cells that displayed greater fluorescence compared with a control staining reaction containing the ALDH inhibitor, DEAB (diethylaminobenzaldehyde). The analysis was performed by using FlowingSoftware 2.0 (Cell Imaging Core, University of Turku, Finland).

### Cell Sorting

ALDEFLUOR kit was used according to the manufacturer's instructions to detect the ALDH activity. Cells were filtered through a 40μM mesh to obtain a single cell suspension. Cell sorting was performed with a MoFLO cell sorter (DAKOCYTOMATION, Fort Collins, Colorado, USA)

### Cell Viability

The SYTOX® Orange Dead Cell Stain (Invitrogen-Molecular Probes, Carlsbad, CA, USA) was used to assess cell viability as per manufacturer's instructions.

### Clonogenic assays

The breast cancer cell lines were grown to 70% confluence and pulse treated with the indicated drugs. 16hrs later, cells were detached(Accutase-GIBCO) and seeded at 500-1500 cells/well into 6-well dishes (Corning-Costar, Tewksbury, MA, USA) in drug-free media. Fresh medium (25%) was added every three days. Colonies were stained with crystal violet (SIGMA) and colonies (>50 cells) counted after 7- 14 days

### NMR sample preparation

Extracellular metabolites were separated by ultrafiltration (AMICON) before methanol/chloroform/water extraction (2/2/1.8) to extract non polar metabolites[28, 29].

### ^1^H-NMR Spectroscopy

For ^1^H-NMR analysis, polar extracts were dissolved in 600μl of 1 mM TSP [(trimethylsilyl)-propionic-2,2,3,3-d_4_ acid] and 10 mM NaN_3_ solution in D_2_O 0.1 M phosphate buffer (pH=7.4), while non-polar extracts were dissolved in 600μl of CDCl_3_ containing 0.03% TMS (tetramethylsilane) / CD_3_OD solution (2:1, v/v).

All 2D ^1^H J-resolved (JRES) NMR spectra were acquired on a 500 MHz DRX Bruker Avance spectrometer (Bruker, Germany) using a double spin echo sequence with 8 transients per increment for a total of 32 increments. These were collected into 16k data points using spectral widths of 6 kHz in F2 and 40 Hz in F1. There was a 3.0 s relaxation delay. The water resonance was suppressed using presaturation. Each FID was Fourier transformed after a multiplication with sine-bell window functions in both dimensions. JRES spectra were tilted by 45°, symmetrised about F1, referenced to TSP at δ_H_ = 0.0 ppm and the proton-decoupled skyline projections (p-JRES) exported using Bruker's XWIN-NMR software. Metabolites were identified using an in-house NMR database and literature data and confirmed by 2D homo- and heteronuclear NMR spectroscopy. ^1^H-NMR spectra pre-processing. All p-JRES exported were aligned and then reduced into spectral bins with widths ranging from 0.01 to 0.02 ppm by using the ACD intelligent bucketing method (ACD/Labs, Canada) that sets the bucket divisions at local minima (within the spectra) to ensure that each resonance is in the same bin throughout all spectra. After this procedure, the ^1^H spectra were divided into n bins. The area within each spectral bin was integrated to yield a n-component vector, where each component value was represented by the integral value corresponding to the specific spectral bin. To compare the spectra, the integrals derived from the bucketing procedure were normalized to the total integral region, following exclusion of bins corresponding to solvent (residual water/HDO, δ4.76-4.82 ppm; CDCl_3_, δ 7.45-7.50 ppm; CD_3_OD,δ 3.33-3.37 ppm), TSP, TMS (δ -0.5-0.5 ppm) and metformin peaks (δ3.03-3.06 ppm). Data from extracellular media were expressed in terms of net balances at 0 and 24hrs. This to establish consumption (c) or production (p) of the selected metabolites in time.

### Univariate statistical data analysis

Analysis of variance (ANOVA) is a technique for analysing experimental data in which one or more (or dependent) variables are measured under various conditions, identified by one or more classification variables. The response is separated into variation attribuTable to differences between the classification variables and variation attribuTable to random errors. An analysis of variance constructs tests to determine the significance of the classification effects. A typical goal is to compare means of the response variables for various combinations of the classification variables (interaction effects).

In our study, we considered metabolite consumption and production as a dependent variable while type of subpopulation, type of cell line and treatment effect as the classification variables. The resulting data was used as input for univariate and multivariate analysis Principal Component Analysis (PCA)[30] and Orthogonal Projections to Latent Structures-Discriminant Analysis (OPLS-DA)[31]. PCA and OPLS-DA were conducted using SIMCA-P+ version 12 (Umetrics, Umea, Sweden).

### Principal Component Analysis

Principal Component Analysis (PCA) is a projection method used for exploiting the information embedded in multidimensional data sets. The data is reduced to a few latent variables (or principal components) collecting the information implicit in the original variables correlation structure. The presence of correlations between the original variables allows for the reduction of dimensionality of the data set in the new space without noticeable loss of information. The extracted components (PCs) are each orthogonal and ordered in terms of percentage of explained variation, with the first components collecting the ‘signal’ (correlated) portion of information, while minor components can be considered as ‘noise’ components. Because the principal components are, by construction, orthogonal to each other, a clear-cut separation of the different and independent features characterizing the data set is made possible. Each statistical unit is assigned a score relative to each extracted component, while the correlation coefficient between each original variable and extracted components (loading) gives a meaning to the PCs. The output from the PCA analysis consists of score plots, which provide an indication of the differences between the classes in terms of metabolic similarity, and loading plots. These loading plots give an indication of which metabolite net balances are important with respect to the classification obtained in the score plots.

### Orthogonal projections to latent structures discriminant analysis (OPLS-DA)

Orthogonal projections to latent structures discriminant analysis (OPLS-DA) is a supervised pattern recognition technique widely used in the field of metabolomics to interpret large multivariate data sets describing differences between the groups under study in a straightforward and accurate way.

OPLS-DA separates the systematic variation in the matrix X (spectroscopic data) into two parts, one linearly related (variation of interest) to the matrix Y (the classification variables) and one orthogonally related (so called orthogonal variation or structured noise) to the matrix Y. This partitioning of the X-data improved the interpretation of the model.

In our study, we considered consumption and production of metabolites as X matrix and type of subpopulation as Y matrix. The influence of the original variables on the obtained model was determined using loading values.

### RNA processing and hybridization

Total RNA from the FACS sorted ALDH^bright^ and ALDH^low^ cells (98 ± 0.4% and 97 ± 1.4% purity, respectively, as assessed by re-FACS) was extracted by TRI Reagent lysis reagent (Life Technologies-AMBION Carlsbad, CA, USA) according to the manufacturer's instructions. The microRNA Complete Labeling and Hyb Kit (AGILENT Santa Clara, CA, USA) was used to generate fluorescent miRNA, according to manufacturer's instructions. Scanning and image analysis were performed using the Agilent DNA Microarray Scanner (P/N G2565BA). Feature Extraction Software (V-10.5) was used for data extraction from raw microarray image files using the miRNA_105_Dec-09FE protocol.

### Analysis of microRNA expression

Pearson's correlation coefficient was calculated in order to assess quality of replicates. ANOVA test α=5%) was performed to assess statistical significance of the observed differences in microRNA profiling (Fig.S2).

### Preprocessing

The signal of 851 human miRNAs was processed by MATLAB (The MathWorks Inc.) in in house-built routines. All background-subtracted intensity values ^low^er than 1 were considered be^low^ detection and thresholded to 1. The arrays were quantile-normalized forcing each slide to assume the same mean distribution and log2-trasformed.

Feature selection. Clusters in data were identified by unsupervised Hierarchical Clustering and Principal Component Analysis. A permutation t-test and bootstrap test were used to select deregulated miRNAs after treatment. A false discovery rate (FDR) procedure was also applied for multiple comparisons. All tests were two-tailed and considered significant if both p-value and q-value (FDR) were less than 0.05.

### miRNA-pathway assignment prediction

A list of predicted miRNAs in pathway experimentally identified from metabolomics was extracted using miRWalk (http://www.umm.uni-heidelberg.de/apps/zmf/mirwalk/)[32]. Predicted miRNAs with significant modulation of the signal after treatment in the array experiment were considered for further investigations.

### cDNA synthesis and gene expression

The first-strand cDNA was synthesized according to manufacturer's instructions (M-MLV Reverse Transcriptase, Life Techonlogies). Gene expression was measured by real-time PCR using the FAST SYBRGreen master mix (Applied Biosystems, Foster City, CA, USA) on a StepOne instrument (Applied Biosytems).

Sequences of the Q-PCR primers are: IRS-2 Fw: ACGCCAGCATTGACTTCTTGT, Rv: GCCAGACAGATCTTCACTCTTTCA; ACTIN Fw: GGCATGGGTCAGAAGGATT, Rv: CACACGCAGCTCATTGTAGAAG; C-MYC Fw: CTCCTGGCAAAAGGTCAGAG, Rv: TCGGTTGTTGCTGATCTGTC.ACTIN was used as endogenous control

### Statistical Analysis

Generally, Student's t-test was used to assess significance of the data generated except where otherwise specified.

## SUPPLEMENTARY FIGURES AND TABLES




