# Supplementary material for: Metformin-induced metabolic reprogramming of chemoresistant ALDHbright breast cancer cells
Source: Oncotarget. 2014 Mar 26;5(12):4129–43. doi: 10.18632/oncotarget.1864 (PMC4147311; doi:10.18632/oncotarget.1864)
Supplement: Supplementary file 1 [file oncotarget-05-4129-s001.pdf]

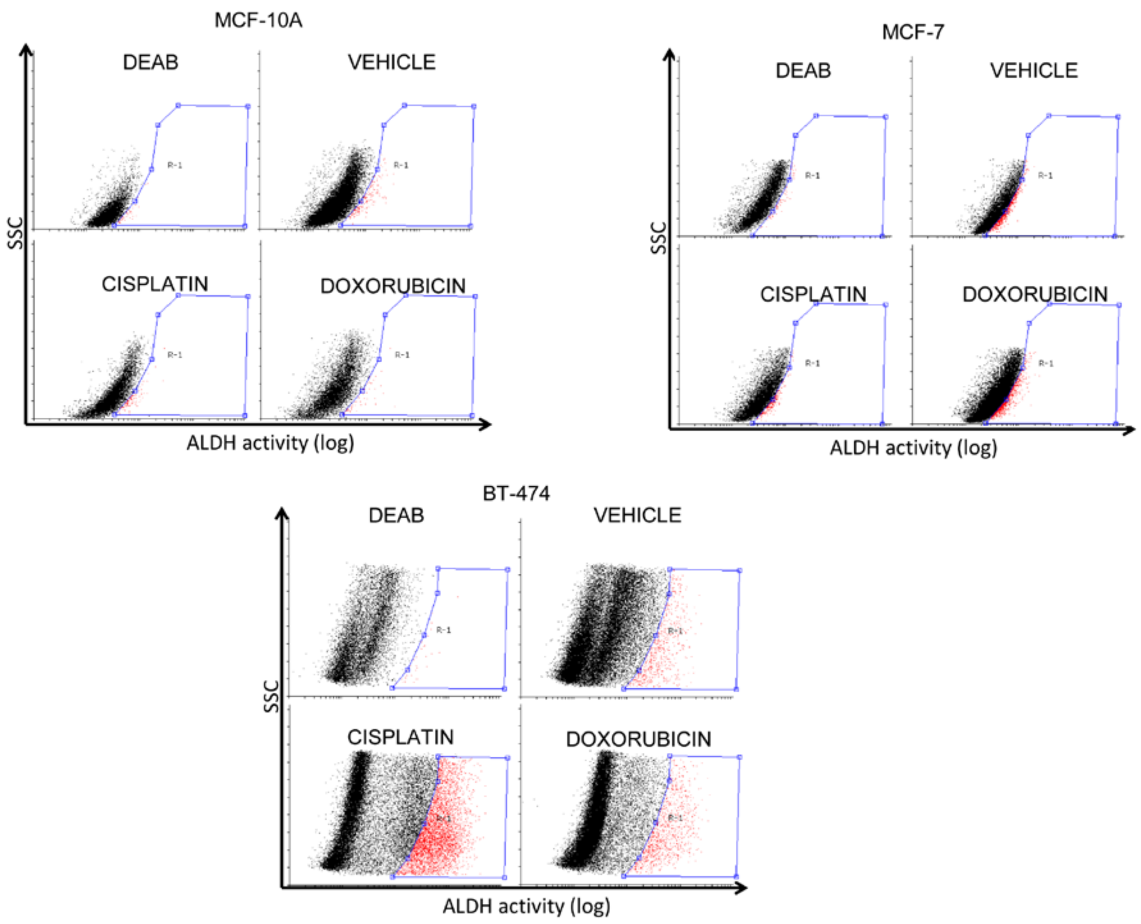

Suppl. Fig.1 . Representative dot plots of MCF10A (A), MCF-7(B) and BT-474(C) cells assayed for Aldhehyde Dehydrogenase activity. Cells enriched for ALDH activity (ALDHbright) were gated (red-r1). To set the background, the percentage of ALDH bright cells was determined in the presence of DEAB, an inhibitor of the ALDH enzyme (Upper left plot for each panel). Please note that a strong decrease of the ALDHbright cells was observed in the cisplatin and doxorubicin-treated MCF-10A cells. The dot plots refer to the values reported in fig. 1B.

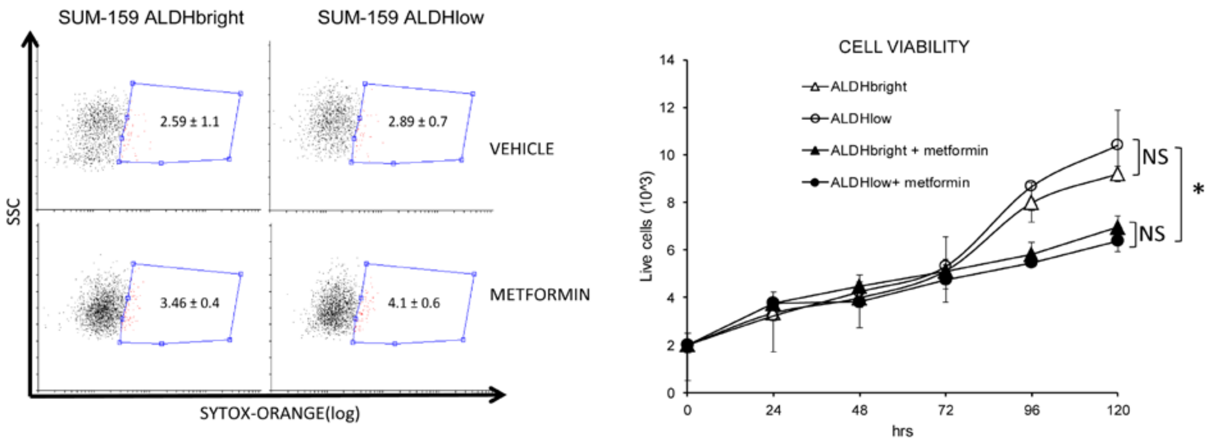

Suppl. Fig. 2. Metformin treatment does not significantly affects the viability of the SUM-159 PT ALDHbright and ALDHlow cells. A. ALDHbright and ALDHlow cells were allowed to recover for 24hrs after sorting and then treated with vehicle or metformin (0.5mM) for 24 hrs. Representative dot plots of the cells stained with the cell impermeant dye Sytox-Orange. The gated (red) cells are apoptotic. Mean ± SE of two independent experiments were reported. **B. Proliferation assays**. 24hrs after sorting, the sorted cells were treated for 120 hrs with vehicle or metformin and cell number assessed any other day by Trypan Blue exclusion dye. Mean ± SE of two independent experiments were reported. NS: not significant *: $p<0.05$ (between vehicle and metformin-treated cells, respectively). Please note that differences in cell number between vehicle and metformin-treated samples were statistically significant after 96hrs treatment . No statistically significant differences between ALDHbright and ALDHlow cells were observed at any time point (NS).
