# Supplementary material for: Metformin-induced metabolic reprogramming of chemoresistant ALDHbright breast cancer cells
Source: Oncotarget. 2014 Mar 26;5(12):4129–43. doi: 10.18632/oncotarget.1864 (PMC4147311; doi:10.18632/oncotarget.1864)
Supplement: Supplementary file 2 [file oncotarget-05-4129-s002.pdf]

Suppl. Table 1. Sources of variability in metabolomic data of ALDH populations from BT-474, MCF-7 and SUM-159-PT cell lines as measured by two-way analysis of variance. The table reports the F-values for each factor (Subpopulation and line) and combination of factors (Subpopulation*line) and, in parenthesis, the corresponding *p*-values. Significant values (p<0.05) are in bold.

	**Factor**		
[a]**PCs**	**Subpopulation**	**Line**	**Subpopulation*Line**
**PC1** (21)	35.54 (**<0.0001**)	98.42 **(<0.0001**)	20.76 (**0.0004**)
**PC2** (13)	1.66 (0.21)	432.77 (**<0.0001**)	4.23 (0.06)
**PC3** (7)	0.20 (0.66)	0.78 (0.39)	18.39 (**0.0007**)
**PC4** (6)	28.08 (**0.0001**)	0.21 (0.65)	0.0004 (0.99)
**PC5** (5)	1.76 (0.21)	0.020 (0.89)	2.91 (0.11)
**PC6** (5)	0.02 (0.89)	0.057 (0.82)	0.02 (0.89)

[a]The data refer to the same samples of conditioned media shown in Figure 2. In parentheses the percent of variance explained by each principal component is reported.

Suppl. Table 2. Sources of variability in metabolomic data of Metformin-treated and untreated breast cancer cell lines as measured by two-way analysis of variance. The table reports the F-values for each factor (subpopulation, line and treatment) and combination of factors (subpopulation*line, subpopulation*treatment and line*treatment) and, in parenthesis, the corresponding *p*-values. Significant values (p<0.05) are in bold.

	**Factor**					
**PCs**	**Subpopulation**	**Line**	**Treatment**	**Subpopulation *Line**	**Subpopulation *Treatment**	**Line *Treatment**
**PC1** (20.8)	89.76 (**<0.0001**)	2055.78 (**<0.0001**)	38.37 (**<0.0001**)	8.06 (**0.001**)	2.54 (0.12)	22.06 (**<0.0001**)
**PC2** (13.1)	0.035 (0.85)	340.0 (**<0.0001**)	17.60 (**0.0002**)	0.16 (0.84)	0.61 (0.44)	2.93 (0.07)
**PC3** (4.9)	0.32 (0.58)	1.35 (0.27)	2.45 (0.13)	0.46 (0.63)	0.27 (0.61)	2.11 (0.14)
**PC4** (4.1)	16.16 (**0.0003**)	0.12 (0.89)	1.44 (0.24)	1.18 (0.32)	6.74 (**0.014**)	0.11 (0.90)
**PC5** (3.9)	3.19 (0.084)	0.53 (0.59)	0.037 (0.85)	4.02 (**0.03**)	0.17 (0.69)	7.82 (**0.002**)
**PC6** (3.7)	21.65 (**<0.0001**)	0.36 (0.70)	8.15 (**0.01**)	2.30 (0.12)	0.19 (0.67)	0.44 (0.65)

Suppl. Table 3. PCA approach was used to quantify the different metabolic effects induced by metformin on each cell line within each subpopulation. Given that component scores are normalized, we can immediately measure the treatment effect on the metabolic components by examining the average difference in each PC score between control and treated groups.

**Average differences in PCs scores between untreated and treated groups[a, b]**			
**Subpopulation**	**Cell line**		
	**BT-474**	**MCF-7**	**SUM-159-PT**
**Aldh^{bright}**	0.86 PC1***<br>0.26 PC2*<br>0.68 PC3**	1.05 PC2**	0.57 PC1*<br>0.90 PC3*<br>0.54 PC4*
**Aldh^{low}**	0.80 PC1***<br>0.65 PC2*<br>0.51 PC3*	0.33 PC1**<br>0.64 PC2***	0.38 PC1**<br>0.82 PC2**

[a]Statistics: *, $p < 0.05$; **, $p < 0.01$; ***, $p < 0.001$.
[b] Variances explained (%) by each PC for BT-474: 32.6, PC1; 15.4, PC2; 12.7, PC3; MCF-7: 24.8, PC1; 14.4, PC2; 12.7, PC3; and SUM-159-PT: 19.3, PC1; 14.1, PC2; 12.3, PC3; 8.95 PC4.

Suppl. Table 4. MicroRNAs modulated by metformin in ALDHbright cells and predicted to modulate metabolic processes.

miR	p-value	FDR	FOLDS (METFORMIN/VEHICLE)
'hsa-miR-193a-3p'	1.72908E-05	0.0007	2.299500589
'hsa-miR-145'	0.033048881	0.0188	2.091334249
'hsa-miR-654-3p'	0.06209168	0.0209	2.045279319
'hsa-miR-450a'	0.020811183	0.0183	2.033280525
'hsa-miR-518b'	0.043814682	0.02	1.949562208
'hsa-miR-335'	0.00349312	0.0097	1.751394795
'hsa-miR-34b'	0.009725852	0.0126	1.718546521
'hsa-miR-1233'	0.053953301	0.0197	1.715973047
'hsa-miR-582-5p'	0.00217658	0.0094	1.52885658
'hsa-miR-551b'	0.006537997	0.0115	1.495632221
'hsa-miR-424'	0.003271108	0.0098	1.493777385
'hsa-miR-32'	0.015882884	0.0154	1.492558488
'hsa-miR-885-5p'	0.014925563	0.0152	1.380905842
'hsa-miR-125b'	0.036365963	0.0201	1.344093959
'hsa-miR-33a'	0.001749666	0.0097	1.275494979
'hsa-miR-452'	0.002723301	0.0106	1.141236674
'hsa-miR-502-5p'	0.063837672	0.021	1.100478721
'hsa-miR-10a'	0.015720612	0.0156	1.078910924
'hsa-miR-219-5p'	0.040290306	0.0193	1.02589016
'hsa-miR-423-3p'	0.040150543	0.0195	0.968052274
'hsa-let-7a'	0.047259606	0.0204	0.932759679
'hsa-miR-28-5p'	0.069295146	0.0215	0.885554084
'hsa-miR-15a'	0.023823863	0.0192	0.880244296
'hsa-miR-101'	0.028863371	0.0193	0.873061301
'hsa-miR-497'	0.01387044	0.0145	0.866189274
'hsa-miR-204'	0.060126228	0.0206	0.865726685
'hsa-miR-96'	0.026546367	0.0194	0.859507725
'hsa-miR-99a'	0.024906435	0.0193	0.853165043
'hsa-miR-27b'	0.059508953	0.0206	0.833342472
'hsa-miR-590-5p'	0.012543805	0.0143	0.82795686
'hsa-miR-196a'	0.051119499	0.0202	0.803946409
'hsa-miR-195'	0.019844403	0.0179	0.799585346
'hsa-miR-513b'	0.066550705	0.021	0.759951113
'hsa-miR-636'	0.010887772	0.0132	0.748374202
'hsa-let-7i'	0.067032631	0.021	0.740827033
'hsa-miR-19a'	0.044387166	0.02	0.739374974
'hsa-miR-652'	0.06530499	0.0209	0.727199793
'hsa-miR-29c'	0.028234483	0.0196	0.726007785
'hsa-miR-95'	0.007914757	0.0128	0.712324432
'hsa-miR-374a'	0.04798183	0.02	0.701409944
'hsa-miR-483-3p'	0.012725072	0.0141	0.700801441
'hsa-miR-301b'	0.02132265	0.0184	0.68428587
'hsa-miR-218'	0.049626295	0.0203	0.678910451
'hsa-let-7e'	0.06417458	0.0209	0.666640218
'hsa-let-7d'	0.054748369	0.0198	0.644274199
'hsa-miR-30c'	0.062658008	0.0209	0.604566364
'hsa-miR-199a-5p'	0.038800918	0.0203	0.595103936
'hsa-miR-26b'	0.056878328	0.0199	0.588673287
'hsa-miR-30b'	0.061871286	0.021	0.564839876
'hsa-miR-181d'	0.066494205	0.0211	0.541714276
'hsa-miR-615-3p'	0.051650405	0.0202	0.52296564
'hsa-miR-30a'	0.053436025	0.0199	0.485673427
'hsa-miR-215'	0.025558584	0.0194	0.477589527
'hsa-miR-212'	0.016736027	0.0158	0.427471657
'hsa-miR-296-5p'	0.04375204	0.0202	0.387219288
'hsa-miR-933'	0.026987091	0.0194	0.371913226
'hsa-miR-362-3p'	0.04807362	0.0198	0.316333479
'hsa-miR-149'	0.036440138	0.0199	0.306483875
'hsa-miR-766'	0.053471888	0.0197	-0.353785877
'hsa-miR-498'	0.050188845	0.0203	-0.360510599
'hsa-miR-126'	0.039784223	0.0195	-0.431207032
'hsa-miR-557'	0.042004591	0.0196	-0.564910718
'hsa-miR-520b'	0.009050624	0.013	-0.631050932
'hsa-miR-520d-3p'	0.047275919	0.0201	-0.701072482
'hsa-miR-202'	0.031141735	0.0198	-0.764664384
'hsa-miR-765'	0.011572416	0.0136	-0.805947985
'hsa-miR-520e'	0.00046923	0.0061	-0.896298282
'hsa-miR-542-5p'	0.037767714	0.0203	-0.911774343
'hsa-miR-28-3p'	0.013400111	0.0144	-0.932751089
'hsa-miR-622'	0.000688593	0.0067	-0.947243836
'hsa-miR-596'	0.004034736	0.0104	-0.960409504
'hsa-miR-892b'	0.032264147	0.019	-0.991730424
'hsa-miR-632'	0.022222761	0.0187	-1.066299144
'hsa-miR-501-5p'	0.046968288	0.0205	-1.070383897
'hsa-miR-610'	0.005709397	0.0117	-1.071418306
'hsa-miR-139-3p'	0.047430156	0.02	-1.087406545
'hsa-miR-148a'	0.001967744	0.0095	-1.178274255
'hsa-miR-422a'	0.039409904	0.0204	-1.361276974
'hsa-miR-483-5p'	0.004481466	0.0109	-1.488609843
'hsa-miR-654-5p'	0.003082586	0.01	-1.591501248
'hsa-miR-760'	0.010656323	0.0133	-1.717811692
'hsa-miR-511'	0.001325285	0.0103	-1.779146234
'hsa-miR-411'	0.029771444	0.0196	-2.020423971
'hsa-miR-601'	0.001484914	0.0096	-2.086084918
'hsa-miR-34c-3p'	0.024256023	0.0192	-2.36032882
'hsa-miR-489'	0.045632523	0.0201	-2.68068956
'hsa-miR-556-5p'	0.06366231	0.0211	-3.083742685
'hsa-miR-571'	0.018481195	0.0171	-3.204801586
'hsa-miR-518d-3p'	0.000252722	0.0049	-3.395393435
